# Supplementary material for: Body Fat Percentage and Availability of Oral Food Intake: Prognostic Factors and Implications for Nutrition in Amyotrophic Lateral Sclerosis
Source: Nutrients. 2021 Oct 21;13(11):3704. doi: 10.3390/nu13113704 (PMC8622757; doi:10.3390/nu13113704)
Supplement: Supplementary file 1 [file nutrients-13-03704-s001.zip › nutrients-1380110-supplementary.pdf]

Table S1. Demographics of ALS patients.

Variables	*n*	Mean ± SD	Median
Gender (M: F)	52 (31:22)		
Age at symptom onset	52	61.25 ± 10.24	62
Age at initial BIA measurement	52	65.04 ± 8.42	65
ΔWeight (g)/duration (month)	52	18.02 ± 946.45	0
ΔMuscle mass (g)/duration (months)	52	328.65 ± 1673.16	-44.93
ΔBody mass index (BMI, kg/m^2)/duration (months)	52	0.01 ± 0.33	0
ΔSkeletal muscle mass (g)/duration (months)	52	136.13 ± 880.04	-38.26
ΔFat-free muscle mass (g)/duration (months)	52	309.32 ± 1599.87	-44.51
ΔSMI (kg/m^2)/duration (months)	52	0.1 ± 0.61	-0.03
ΔBody fat percentage (%)/duration (months)	52	-0.77 ± 4.66	0.1
ΔPhase angle (degree)/duration (months)	52	-0.16 ± 0.31	-0.09
Δ ECW/TBW (mL)/duration (months)	52	2.19 ± 4.87	1.09
ΔBasal metabolism (kcal)/duration (months)	52	6.73 ± 34.73	-0.89
Duration between measurement (months)	52	7.46 ± 5.08	6.17
Number of patients with tracheostomy	25		
Number of patients with possible oral food intake	11		
Number of patients with PEG	15		
Number of patients with a nasogastric tube	25		

Δ = subtraction from follow-up measurements to baseline BIA values. SD = standard deviation.
